# Structure–activity relationship and mechanism of four monostilbenes with respect to ferroptosis inhibition†

**DOI:** 10.1039/d0ra04896h

**Published:** 2020-08-21

**Authors:** Xiaojian Ouyang, Xican Li, Jie Liu, Yangping Liu, Yulu Xie, Zhongcun Du, Hong Xie, Ban Chen, Wenbiao Lu, Dongfeng Chen

**Affiliations:** School of Chinese Herbal Medicine, Guangzhou University of Chinese Medicine Guangzhou 510006 China lixican@126.com; School of Basic Medical Science, Guangzhou University of Chinese Medicine Guangzhou 510006 China chen888@gzucm.edu.cn; The Research Center of Basic Integrative Medicine, Guangzhou University of Chinese Medicine Guangzhou 510006 China

## Abstract

Erastin-treated bone marrow-derived mesenchymal stem cells (bmMSCs) were prepared and used to compare the ferroptosis inhibitory bioactivities of four monostilbenes, including rhapontigenin (1a), isorhapontigenin (1b), piceatannol-3′-*O*-glucoside (1c), and rhapontin (1d). Their relative levels were 1c ≈ 1b > 1a ≈ 1d in 4,4-difluoro-5-(4-phenyl-1,3-butadienyl)-4-bora-3*a*,4*a*-diaza-*s*-indacene-3-undecanoic acid (C11-BODIPY), 3-(4,5-dimethylthiazol-2-yl)-2,5-diphenyltetrazolium bromide (MTT), and flow cytometric assays. The comparison highlighted two 4′-OH-containing monostilbenes (1c and 1b) in ferroptosis inhibitory bioactivity. Similar structure–activity relationships were also observed in antioxidant assays, including 1,1-diphenyl-2-picryl-hydrazl radical (DPPH˙)-trapping, 2-phenyl-4,4,5,5-tetramethylimidazoline-1-oxyl 3-oxide radical (PTIO˙)-trapping, and Fe^3+^-reducing assays. UPLC-ESI-Q-TOF-MS analysis of the DPPH˙-trapping reaction of the monostilbenes revealed that they can inhibit ferroptosis in erastin-treated bmMSCs through a hydrogen donation-based antioxidant pathway. After hydrogen donation, these monostilbenes usually produce the corresponding stable dimers; additionally, the hydrogen donation potential was enhanced by the 4′-OH. The enhancement by 4′-OH can be attributed to the transannular resonance effect. This effect can be used to predict the inhibition potential of other π–π conjugative phenolics.

## Abstract 2

Erastin-treated bone marrow-derived mesenchymal stem cells (bmMSCs) were prepared and used to compare the ferroptosis inhibitory bioactivities of four monostilbenes, including rhapontigenin (1a), isorhapontigenin (1b), piceatannol-3′-*O*-glucoside (1c), and rhapontin (1d).

## Graphical abstract

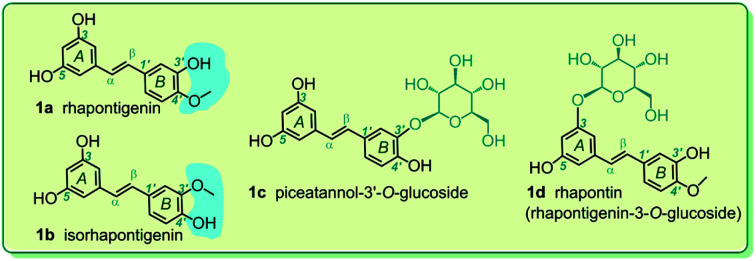

## Introduction

Ferroptosis is a newly described form of apoptosis that depends on the accumulation of Fe^2+^.^1,2^. The accumulated Fe^2+^ catalyzes the generation of numerous lipid peroxides (LPOs), thereby causing cell death.^3–5^ Promotion of ferroptosis is considered a novel strategy for treating cancer as ferroptosis can suppress tumor cell growth.^6–8^

In contrast, inhibition of ferroptosis can improve the viability of normal cells, such as bone marrow-derived mesenchymal stem cells (bmMSCs). Improved viability of bmMSCs ensures enough living seed cells for transplantation for treating various diseases, such as Parkinson's, Alzheimer's, and aging diseases.^9–13^ Thus, searching for novel ferroptosis inhibitors has become a major focus in recent years.^11,14^

Some radical-trapping antioxidants (*e.g.*, baicalein) have been suggested as ferroptosis inhibitors. In addition, some reductants with electron-donating potential could also function as ferroptosis inhibitors.^14,15^ As radical trapping and electron donation are the characteristics of monostilbenes, they can be considered natural antioxidants.^16–19^ This implies that monostilbenes may have the potential to inhibit ferroptosis.

**Fig. 1:**
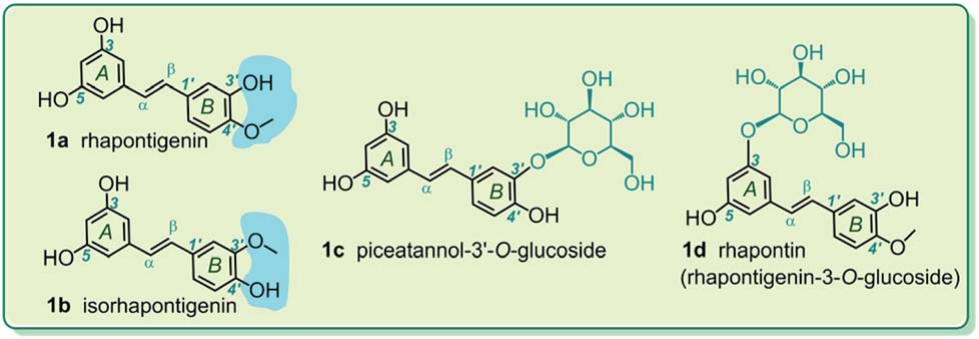
The structures of rhapontigenin (1a), isorhapontigenin (1b), piceatannol-3′-*O*-glucoside (1c), and rhapontin (rhapontigenin-3-*O*-glucoside, 1d).

Based on the implication that monostilbenes can serve as potential inhibitors of ferroptosis, four monostilbenes were selected as the model compounds in this study, including rhapontigenin (1a), isorhapontigenin (1b), piceatannol-3′-*O*-glucoside (1c), and rhapontin (1d). These monostilbenes naturally occur in a number of medicinal plants, such as *Stuhlmannia moavi*, *Gnetum hainanense*, and *Gnetum parvifolium*.^20–24^ As seen in Fig. 1, all these monostilbenes were constructed using a *trans*-1,2-diphenylethene skeleton, which comprises the π–π conjugative system. Though their structures were similar, all the monostilbenes differed from one another. Rhapontigenin (1a) and isorhapontigenin (1b) consist of a pair of positional isomers. Rhapontin (1d) is the glucoside of rhapontigenin (1b). Therefore, comparison of their structures can be used to analyze the structure–activity relationship in the ferroptosis inhibitory action of the monostilbene family.

As potential ferroptosis inhibitors their mechanisms of action should be extensively studied, especially as during the inhibition of ferroptosis, the monostilbenes are inevitably exposed to various free radicals, including LPOs, reactive oxygen species (ROS),^25^ and even reactive nitrogen species (RNS),^26,27^ which can oxidize monostilbenes into unstable radical intermediates by virtue of their strong hydrogen donation ability. Usually, these unstable radical intermediates are reactive and can cause oxidative damage to the seed cells.^28,29^

To explore their underlying mechanisms further, these monostilbenes were incubated with 1,1-diphenyl-2-picryl-hydrazl radical (DPPH˙) and analyzed using cutting-edge ultra-performance liquid chromatography coupled with electrospray ionization quadrupole time-of-flight tandem mass spectrometry (UPLC-ESI-Q-TOF-MS) technology. The mechanism study will be helpful to judge the possible oxidative damage caused by the radical intermediates arising from monostilbenes, and it would aid the clinical application of monostilbenes during stem cell transplantation. The outcome of the structure–activity relationship analysis could help medicinal chemists to design more effective ferroptosis inhibitors.

## Materials and methods

### Animals, biological kits, and chemicals

Four-week-old Sprague-Dawley (SD) rats were obtained from the Animal Center of Guangzhou University of Chinese Medicine (Guangzhou, China). All animal procedures were performed in accordance with the Guidelines for Care and Use of Laboratory Animals of Guangzhou University of Chinese Medicine; the experiments were approved by the Animal Ethics Committee of Guangzhou University of Chinese Medicine (Guangzhou, China). The complete medium with glucose for SD rat bone marrow (bmMSCs) was purchased from Cyagen Biosciences (CA, USA). Dulbecco's modified Eagle's medium, fetal bovine serum, and trypsin were obtained from Molecular Probes (Carlsbad, CA, USA). 3-(4,5-Dimethylthiazol-2-yl)-2,5-diphenyltetrazolium bromide (MTT) was obtained from Sigma-Aldrich Shanghai Trading Co. An Annexin V/propidium iodide (PI) assay kit was purchased from BD Biosciences (NJ, USA). The probe C11-BODIPY was purchased from Molecular Probes (CA, USA). Erastin was obtained from MedChemExpress (Monmouth Junction, NJ, USA). Ferrostatin-1 (Fer-1) was purchased from Selleck Chemicals (Houston, TX, USA).

Rhapontigenin (C_15_H_14_O_4_, CAS number: 500-65-2, MW: 258.3, purity 98%), isorhapontigenin (C_15_H_14_O_4_, CAS number: 32507-66-7, MW: 258.3, purity 98%), piceatannol-3′-*O*-glucoside (C_20_H_22_O_9_, CAS number: 94356-26-0, MW: 406.4, purity 98%), and rhapontin (C_21_H_24_O_9_, CAS number: 155-58-8, MW: 420.4, purity 98%) were obtained from Chengdu Biopurify Phytochemicals, Ltd. (Chengdu, China). (±)-6-Hydroxyl-2,5,7,8-tetramethylchromane-2-carboxylic acid (Trolox) were obtained from Sigma-Aldrich (Shanghai, China). DPPH˙ (C_18_H_12_N_5_O_6_) was obtained from Aladdin Chemical Ltd. (Shanghai, China). 2,4,6-Tripyridyltriazine (TPTZ) was obtained from Sigma-Aldrich (Shanghai, China). The 2-phenyl-4,4,5,5-tetramethylimidazoline-1-oxyl-3-oxide radical (PTIO˙) was obtained from TCI Chemical Co. (Shanghai, China). Methanol and the other reagents were purchased from Guangdong Guanghua Chemical Plants Co., Ltd. (Shantou, China).

### Prevention of erastin-treated ferroptosis in bmMSCs

The erastin-induced ferroptosis model of bmMSCs was created based on recent studies^30,31^ with modifications. To measure the anti-ferroptosis bioactivities of four monostilbene, three assays were used, C11-BODIPY assay, MTT assay and flow cytometry.

The C11-BODIPY assay was used to characterize the degree of lipid peroxidation.^32,33^ In brief, the cultured bmMSCs were seeded at 1 × 10^3^ cells per well in 24-well plates. After adherence for 24 h, the bmMSCs were divided into control, model, and sample groups. In the control group, bmMSCs were incubated for 12 h in Stel Basal medium. In the model and sample groups, bmMSCs were incubated in the presence of erastin (20 μM). After incubation for 12 h, the mixture of erastin and medium was removed. The bmMSCs in the model group were incubated for 12 h in Stel Basal medium, while bmMSCs in the sample group were incubated for 12 h in Stel Basal medium with the monostilbene samples. The concentration of the four monostilbene samples was 100 μM. The fluorescence of the incubated cells was determined using the fluorescent probe C11-BODIPY (Invitrogen, Molecular Probes). Cells were incubated for 30 min prior to analysis with C11-BODIPY (2.5 μM). Photos were taken under a fluorescence microscope.

The MTT assay was performed by the methods presented in previous studies^34–36^ with minor modifications. In brief, the cultured bmMSCs were seeded at 1 × 10^4^ cells per well in 96-well plates. After adherence for 12 h, the bmMSCs were divided into control, erastin, erastin plus Fer-1, and sample groups. The model and sample groups were added by 20 μM erastin for 12 h; the erastin plus Fer-1 group was added with 20 μM erastin and 1 μM Fer-1. After 12 h, erastin and Fer-1 were removed; the erastin group and erastin plus Fer-1 group were incubated in Stel Basal medium while the sample groups were further divided into 1, 10, 100, and 1000 μM groups, and incubated with various drugs and concentrations. After 12 h, all these groups were added by 20 μL per well MTT (5 mg mL^−1^). The culture was incubated for an additional 4 h, then the culture medium was discarded and 150 μL DMSO per well was added for 10 min. Absorbance was measured at 490 nm on a BioKinetics reader. According to the *A*_490 nm_ values, viability was calculated.

The flow cytometric assay was conducted according to the methods proposed in previous studies.^36^ In brief, the cultured bmMSCs were seeded at 1 × 10^6^ cells per well in six-well plates. They were washed twice with cold phosphate-buffered saline then resuspended in 1× binding buffer at a concentration of 1 × 10^6^ cells per mL. Then, 100 μL of the solution (1 × 10^5^ cells) was transferred to a 5 mL culture tube, and 5 μL of fluorescein isothiocyanate (FITC) Annexin V and 5 μL PI was added. The cells were gently vortexed and incubated for 15 min at room temperature in the dark, and 400 mL of 1× binding buffer was added to each tube. After adherence for 12 h, the bmMSCs were divided into control, erastin (model), erastin plus Fer-1, and sample groups. The concentration of the four monostilbene samples was 30 μM. The three groups were analyzed by flow cytometry. Each sample test was repeated in three independent wells.

### DPPH˙ radical-trapping analysis

DPPH˙ radical trapping was determined as previously described with minor modifications.^37,38^ Briefly, 80 μL of DPPH˙ solution (0.1 mol L^−1^) was mixed with methanolic sample solutions at the indicated concentration (*x* = 0–10 μL, 0.5 mg mL^−1^). The mixture was maintained at room temperature, and absorbance was measured at 519 nm on a microplate reader (Multiskan FC, Thermo Scientific, Shanghai, China) against a methanol blank. The percentage of DPPH˙ scavenging activity was calculated as follows:
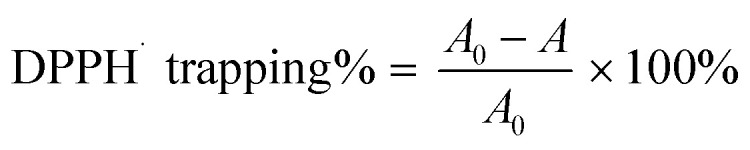
where *A*_0_ is the absorbance of the control without the sample and *A* is the absorbance of the reaction mixture with the sample.

### PTIO˙ radical-trapping analysis

The PTIO˙-scavenging assay was conducted based on a method established by our team.^39^ Briefly, the PTIO˙ radical was dissolved in phosphate buffer (pH 7.4) to prepare a PTIO˙ solution. The samples were prepared using methanol. Various sample volumes were mixed with phosphate buffer and treated with the PTIO˙ solution. After incubation for 1 h at 37 °C, the product mixture was analyzed by measuring the absorbance at 560 nm on microplate reader. The PTIO˙-trapping percentage was calculated based on the formula used for the DPPH˙ radical-trapping assay.

### Fe^3+^-reducing antioxidant power (FRAP) assay

The FRAP assay was adapted from a previously reported method.^40,41^ Briefly, the FRAP reagent was freshly prepared by mixing 10 mM TPTZ, 20 mM FeCl_3_, and 0.25 M pH 3.6 acetate buffer at a volume ratio of 1 : 1 : 10. The test sample (*x* = 0–10 μL, 0.5 mg mL^−1^) was added to (20 − *x*) μL of methanol followed by 80 μL of FRAP reagent. The absorbance was measured at 595 nm after incubating for 30 min at room temperature using the acetate buffer as the blank. The relative reducing power of the sample compared to the maximum absorbance was calculated using the following formula:
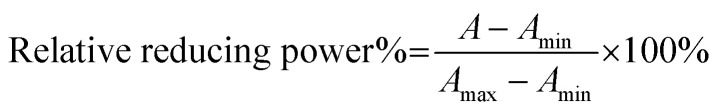
where *A*_max_ is the maximum absorbance at 595 nm and *A*_min_ is the minimum absorbance in the test. *A* is the absorbance of the sample.

### UPLC-ESI-Q-TOF-MS analysis of the dimerization products of the four monostilbenes interacting with DPPH˙

The reaction of DPPH˙ with the four monostilbenes proceeded under the conditions described previously.^42^ In brief, a methanol solution of the four monostilbenes was mixed with a methanol DPPH˙ solution at a molar ratio of 1 : 2, and the resulting mixture was incubated for 24 h at room temperature. Subsequently, the product was passed through a 0.22 μm filter for UPLC-ESI-Q-TOF-MS analysis.

UPLC-ESI-Q-TOF-MS analysis was conducted based on the method described in our previous study.^43^ The UPLC-ESI-Q-TOF-MS analysis system was equipped with a Phenomenex Luna C_18_ column (2.1 mm i.d. × 100 mm, 1.6 μm, Phenomenex Inc., Torrance, CA, USA). The mobile phase was employed for the elution of the system, and consisted of a mixture of methanol (phase A) and 0.1% formic acid water (phase B). The column was eluted at a flow rate of 0.2 mL min^−1^ with the following gradient elution program: 0–2 min, maintained at 25% B; 2–10 min, 30–0% B; 10–12 min, 0–25% B. The sample injection volume was set at 3 μL for the separation of the different components. The Q-TOF-MS analysis was performed on a Triple TOF 5600^plus^ mass spectrometer (AB SCIEX, Framingham, MA, USA) equipped with an ESI source, which was run in the negative ionization mode. The scan range was set at 50–1500 Da. The system was run using the following parameters: ion spray voltage, −4500 V; ion source heater temperature, 550 °C; curtain gas pressure (CUR, N_2_), 30 psi; nebulizing gas pressure (GS1, air), 50 psi; Tis gas pressure (GS2, air), 50 psi. The declustering potential was set at −100 V, whereas the collision energy (CE) was set at −45 V with a CE spread of 15 V.

### Statistical analysis

The results were reported as the mean ± SD of three independent measurements. The IC_50_ values were calculated by linear regression analysis, and independent-sample *t*-tests were performed to compare the different groups.^44^ A *p* value of less than 0.05 was considered significant. The statistical analyses were performed using SPSS software 17.0 (SPSS Inc., Chicago, IL, USA) for windows. All linear regression analyses described in this paper were processed using version 6.0 of Origin professional software (OriginLab Corporation, Northampton, MA, USA).

## Results and discussion

**Fig. 2:**
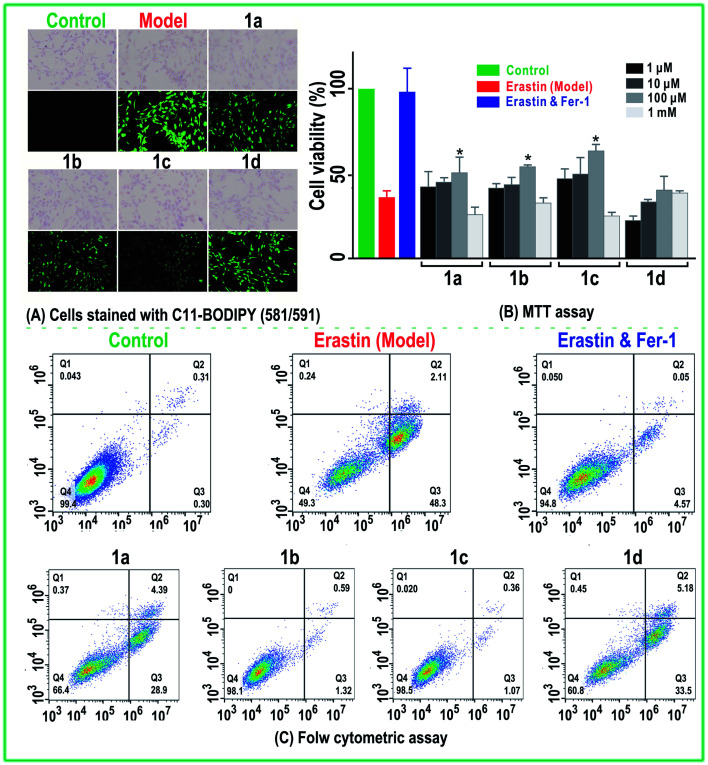
Inhibition of ferroptosis by four monostilbenes in erastin-treated bone marrow-derived mesenchymal stem cells (bmMSCs). (A) C11-BODIPY (4,4-difluoro-5-(4-phenyl-1,3-butadienyl)-4-bora-3*a*,4*a*-diaza-*s*-indacene-3-undecanoic acid) staining assays; (B) 3-(4,5-dimethylthiazol-2-yl)-2,5-diphenyltetrazolium bromide (MTT) assays; (C) flow cytometric assays. 1a, rhapontigenin; 1b, isorhapontigenin; 1c, piceatannol-3′-*O*-glucoside; 1d, rhapontin. Each value is expressed as the mean ± SD, *n* = 3; *: the sample group (100 μM) had a significantly (*p* < 0.05) lower fluorescence intensity than the erastin-treated group.

As seen in Fig. 2A, after staining with C11-BODIPY, the erastin-treated bmMSCs stained dark green in color in the model group, implying that erastin induced LPO accumulation on a large scale.^11,31,45,46^ Correspondingly, the model group displayed low viability (49.3%) in the flow cytometric analysis; this viability was much lower than that in the control group (99.4%) (Fig. 2C), suggesting that the erastin-treated ferroptosis model was successfully created in the bmMSCs.

The bmMSCs model was used to evaluate the levels of ferroptosis inhibition by the four monostilbenes (1a–1d). The cellular viability was analyzed *via* MTT (Fig. 2B) and flow cytometry (Fig. 2C). In MTT assay, the cellular viability of erastin-treated group was significantly (*p* < 0.05) lower than that of the control group. After incubated with 100 μM of three monostilbenes (1a–1c), the cellular viability increased significantly (Fig. 2B). As shown in Fig. 2A, 1c and 1b displayed stronger LPO inhibitory abilities than 1a and 1d. Similarly, cells treated with 1c and 1b showed higher viability percentages than those treated with 1a and 1d in MTT and flow cytometry assays. As shown in Fig. 2C, cells treated with 1a and 1d showed high degree of early apoptosis (28.9% and 33.5%, respectively), and accordingly, exhibited low viability (66.4% and 68.0%, respectively). In comparison, cells treated with 1c and 1b had higher viability (98.1% and 98.5%, respectively). The viability of cells treated with 1c (30 μM) and 1b (30 μM) was generally equivalent to that of cells treated with ferrostin-1 (1 μM) and β-mercaptoethanol (50 μM).^14,47^ The effectiveness of the two monostilbenes (1c and 1b) at inhibiting ferroptosis indicates that they are suitable for use in bmMSCs transplantation during the treatment of neurodegenerative diseases (*e.g.*, Parkinson's disease).^48–50^

The inhibition of ferroptosis has previously been found to be closely related to antioxidant activity,^14^ hence, in this study, the four monostilbenes were comparatively evaluated using three antioxidant assays, including DPPH˙-trapping, PTIO˙-trapping, and Fe^3+^-reducing assays. As shown in Table 1 and Fig. S1–S3,† the four monostilbenes dose-dependently increased the antioxidant levels in these assays. However, their IC_50_ values suggested that relative antioxidant levels were 1c ≈ 1b > 1a ≈ 1d, which could also be found in the ferroptosis inhibition assays. This similarity suggests that ferroptosis inhibition is closely related to antioxidant activity.

**Table 1:** The IC_50_ values (μM) of the four monostilbenes in the antioxidant assays^a^

Antioxidant analyses	DPPH˙-trapping	PTIO˙-trapping	Fe^3+^-reducing
Rhapontigenin (1a)	162.5 ± 4.5^d^	241.9 ± 6.8^c^	240.6 ± 1.8^d^
Isorhapontigenin (1b)	102.5 ± 2.8^c^	188.8 ± 1.7^b^	155.9 ± 5.0^b^
Piceatannol-3′-*O*-glucoside (1c)	92.1 ± 5.5^b^	117.0 ± 6.8^a^	131.3 ± 0.7^b^
Rhapontin (1d)	109.3 ± 0.8^c^	196.1 ± 7.2^b^	208.9 ± 26.5^c^
Trolox	59.7 ± 1.3^a^	124.7 ± 0.5^a^	88.8 ± 1.8^a^

a IC_50_ is defined as the lowest concentration resulting in 50% radical inhibition or relative reducing power. It has been calculated by linear regression analysis and expressed as the mean ± SD (*n* = 3). The linear regression analysis was performed using Origin 6.0 professional software. The IC_50_ values with different superscripts (a, b, c, or d) among the four monostilbenes are significantly different (*p* < 0.05). Trolox was used as the positive control. All dose-dependent curves are provided in Fig. S1–S3.

Among these antioxidant assays, DPPH˙- and PTIO˙-trapping characterized the RNS- and ROS-trapping potentials, respectively because the former is a nitrogen-centered radical and the latter is an oxygen-centered radical. The Fe^3+^-reducing assay is a readout of the electron-donating potential, which affects the antioxidant potential as well as the inhibition of ferroptosis.^14,15,51–53^ However, electron donation is always accompanied by proton donation in cells.^54,55^ The net result of electron and proton donation is generally equivalent to that of whole hydrogen donation.

**Fig. 3:**
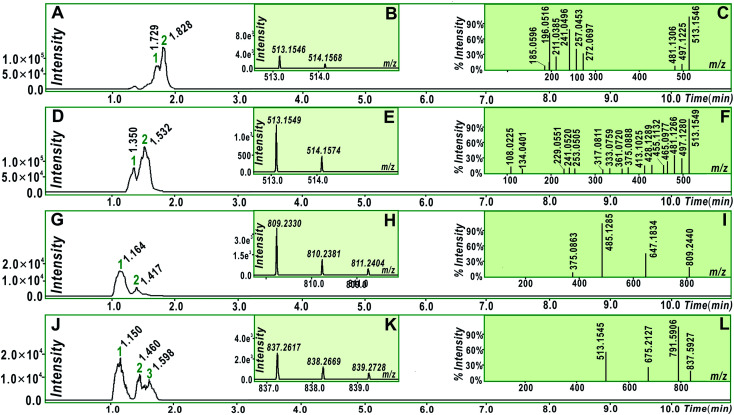
UPLC-ESI-Q-TOF-MS analysis of radical adduct formation dimers of the four monostilbenes interacting with DPPH radicals. (A) Chromatogram of the rhapontigenin dimer when the formula [C_30_H_26_O_8_–H]^−^ was extracted; (B) primary MS spectrum of the rhapontigenin dimer (peak 2); (C) secondary MS spectrum of the rhapontigenin dimer; (D) chromatogram of the isorhapontigenin dimer when the formula [C_30_H_26_O_8_–H]^−^ was extracted; (E) primary MS spectrum of the isorhapontigenin dimer (peak 1); (F) secondary MS spectrum of the isorhapontigenin dimer; (G) chromatogram of the piceatannol-3′-*O*-glucoside dimer when the formula [C_40_H_42_O_18_–H]^−^ was extracted; (H) primary MS spectrum of the piceatannol-3′-*O*-glucoside dimer (peak 1); (I) secondary MS spectrum of the piceatannol-3′-*O*-glucoside dimer; (J) chromatogram of the rhapontin dimer when the formula [C_42_H_46_O_18_–H]^−^ was extracted; (K) primary MS spectrum of the rhapontin dimer (peak 1); (L) secondary MS spectrum of the rhapontin dimer.

To explore whether the four monostilbenes have hydrogen donation potential, they were analyzed using UPLC-ESI-Q-TOF-MS after the DPPH˙-trapping reactions. As shown in Fig. 3, all four monostilbenes yielded the corresponding peaks with double *m*/*z* values. For instance, isorhapontigenin yielded a molecular ion peak ([M] = *m*/*z* 514.1574), along with an [M − H] peak (*m*/*z* 513.1549). The *m*/*z* value (514.1574) was exactly double the MW. value of isorhapontigenin (258.27) and minus the relative mass of two hydrogen atoms; this had only 1.1 × 10^−5^ relative deviation from the calculated value (514.1628). In the present study, molecular weight calculations were conducted based on the accurate relative atomic masses. The relative atomic masses of C, H, O, and N were 12.0000, 1.007825, 15.994915, and 14.003074, respectively.^56^ Therefore, the generation of a isorhapontigenin dimer was identified; this further verified the donation of a hydrogen atom, because if isorhapontigenin had not donated a hydrogen atom, it would not have formed a dimer, according to previous studies.^57–60^

**Fig. 4:**
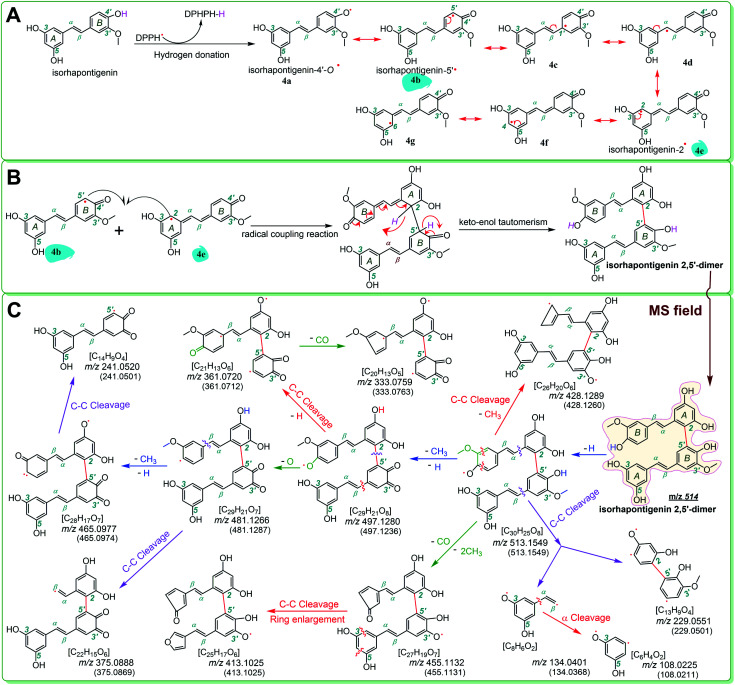
The dimerization reaction of isorhapontigenin (A and B) and MS elucidation (C) (the MS spectra were in the negative ion mode. The accurate *m*/*z* values are shown in Fig. 3 and are rounded to an integer in MS elucidation). Other reasonable fragmenting pathways should not be excluded.

Herein, hydrogen donation is not limited to the typical hydrogen-atom-transfer; other antioxidant pathways could also result in hydrogen donation, such as proton-coupled electron transfer.^54,55,61–63^ The hydrogen donation and subsequent dimerization reactions are shown in Fig. 4, where the isorhapontigenin was linked at 2,5′ in agreement with the results of previous studies^57–67^ and our MS analysis (Fig. 4C).

**Fig. 5:**
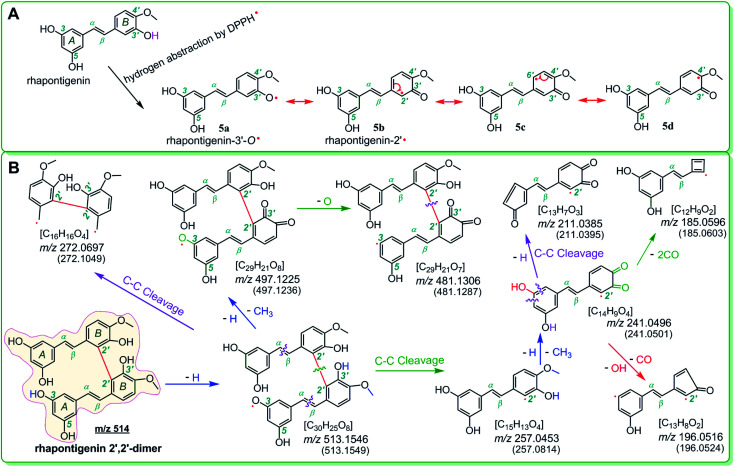
The resonance extreme formula of rhapontigenin (1a) phenoxy radical intermediate and the MS-mediated elucidation of rhapontigenin dimer. Other reasonable fragmenting pathways should not be excluded. Nevertheless, it is impossible to produce a 2,5′-dimer.

Of course, different linking sites might be involved in the formation of dimers by other monostilbenes. The MS of the 2′,2′-rhapontigenin dimer revealed a covalent linkage at the 2′,2′-sites of rhapontigenin (1a) (Fig. 5). However, even if rhapontigenin donated the hydrogen atoms present at 3-OH or 5-OH, it would be impossible to form a 2,5′-dimer. This means that the two monostilbenes isomers (1a and 1b) use different active sites for radical coupling (*i.e.*, herein the dimerization reaction). The difference can only be attributed to the arrangement of phenolic –OH. The difference also highlighted the role of 4′-OH in isorhapontigenin. This is supported by previous studies.^57–60,64–68^

The phenoxy radical of 4′-O˙ (*e.g.*, 4a) possessed more resonance extreme formula of radical intermediate than the phenoxy radical of 3′-O˙ (*e.g.*, 5a) because it can resonate into another ring (*i.e.*, from B-ring to A-ring, Fig. 4A). Therefore, the advantage of 4′-OH in the hydrogen donation reaction can ultimately be attributed to a transannular resonance effect.

The transannular resonance effect is described as follows: in a π–π conjugative phenolic molecule, there is a phenolic –OH that can donate hydrogen to produce a phenoxy intermediate radical; if the radical can be transferred from one ring to another *via* resonance, it might result in enhanced ferroptosis inhibition and antioxidant activities, and the molecule can possibly form a cross-ring dimer.

Product analysis revealed the formation of the isorhapontigenin dimer and rhapontigenin dimer. Similar dimers could also be formed by the other two monostilbenes (1c, 1d) (Fig. S4 and S5†). This indicates that once the monostilbenes are exposed to free radicals (such as LPO, ROS, and RNS), they produce stable dimers and cannot oxidatively damage the cellular biomolecules during the inhibition of ferroptosis.

Due to the transannular resonance effect, another monostilbene, piceatannol-3′-*O*-glucoside (1c), also presented potent bioactivities in cellular assays (Fig. 2), DPPH˙-trapping, PTIO˙-trapping, and Fe^3+^-reducing antioxidant assays (Table 1 and Fig. S1–S3†). Therefore, the relative levels of the four monostilbenes in the ferroptosis inhibition assays correspond to those in antioxidant assays. This further suggests that hydrogen donation plays a central role in ferroptosis inhibition and antioxidant action.

The transannular resonance effect also explains why the bioactivity of rhapontigenin (1a) differs slightly from that of its glucoside rhapontin (1d). Their structural difference relies on the unimportant 3-OH, which cannot result in the generation of the transannular resonance extreme formula after hydrogen donation. Similarly, it also explains why isorhapontigenin (1b) and piceatannol-3′-*O*-glucoside (1c) differ slightly with respect to their bioactivities.

As evidenced in the UPLC-ESI-Q-TOF-MS analysis, the transannular resonance effect can also alter the linkage of dimers. The transannular resonance effect is reflected in the presence of different linkage sites in isorhapontigenin 2,5′-dimer and rhapontigenin 2′,2′-dimer. This implies that the transannular resonance effect can also be used to predict not only the relative antioxidant levels but also antioxidant products generated by members of the monostilbene family. This prediction may also be extrapolated to other phenolic families, including flavonoid, chalcone, and cinnamic acid derivatives (Fig. S6†). This is because these phenolics contain a similar π–π conjugation and arrays of phenolic –OH at various positions.

## Conclusions

The four monostilbenes exhibited an inhibitory effect on ferroptosis by virtue of their 4′-OH, which enhances the inhibitory effect of monostilbenes. This enhancement can be attributed to the hydrogen donation reaction, through which the monostilbenes produce radical intermediates. The 4′-OH-containing monostilbene radical intermediates can transfer an unpaired electron *via* transannular resonance, and thus, become stable. Therefore, transannular resonance effects stabilize the radical intermediates and enhance their ferroptosis inhibition activities.

## Conflicts of interest

There are no conflicts to declare.

## Supplementary Material

RA-010-D0RA04896H-s001
