# Supplementary material for: Structure–activity relationship and mechanism of four monostilbenes with respect to ferroptosis inhibition
Source: RSC Adv. 2020 Aug 21;10(52):31171–9. doi: 10.1039/d0ra04896h (PMC9056428; doi:10.1039/d0ra04896h)
Supplement: RA-010-D0RA04896H-s001 [file RA-010-D0RA04896H-s001.pdf]

Electronic Supplementary Material (ESI) for RSC Advances.
This journal is © The Royal Society of Chemistry 2020

Electronic supplementary information (ESI)

Structure-activity Relationship and Mechanism of Four Monostilbenes with Respect to Ferroptosis Inhibition

Xiaojian Ouyang, [a] Xican Li, [*a] Jie Liu, [bc] Yangping Liu, [bc] Yulu Xie, [a] Zhongcun Du, [a] Hong Xie, [a] Ban Chen, [a] Wenbiao Lu [a] and Dongfeng Chen [*bc]

[a] School of Chinese Herbal Medicine; Guangzhou University of Chinese Medicine, Guangzhou 510006, China. E-mail: lixican@126.com; lixc@gzucm.edu.cn

[b] School of Basic Medical Science, Guangzhou University of Chinese Medicine, Guangzhou 510006, China. E-mail: chen888@gzucm.edu.cn

[c] The Research Center of Basic Integrative Medicine, Guangzhou University of Chinese Medicine, Guangzhou 510006, China

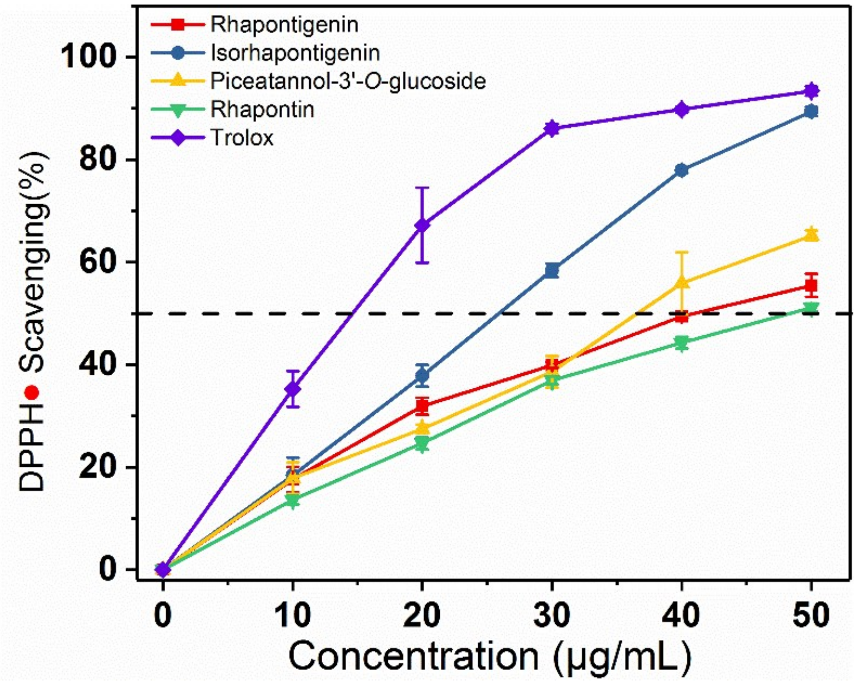

Fig. S1 Dose response curves in the DPPH scavenging spectrophotometric analysis.

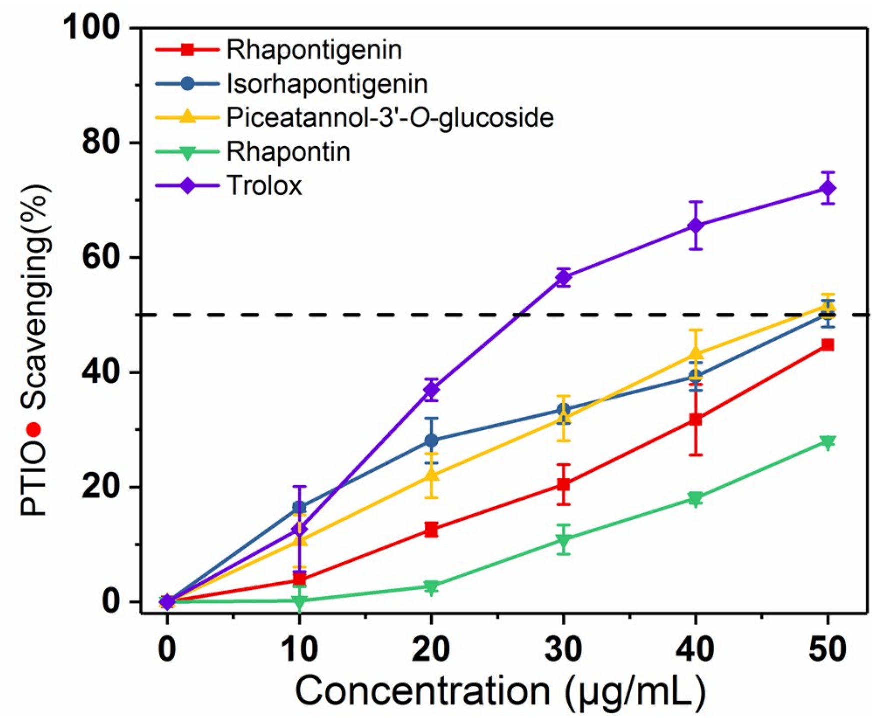

Fig. S2 Dose response curves in the PITO scavenging spectrophotometric analysis.

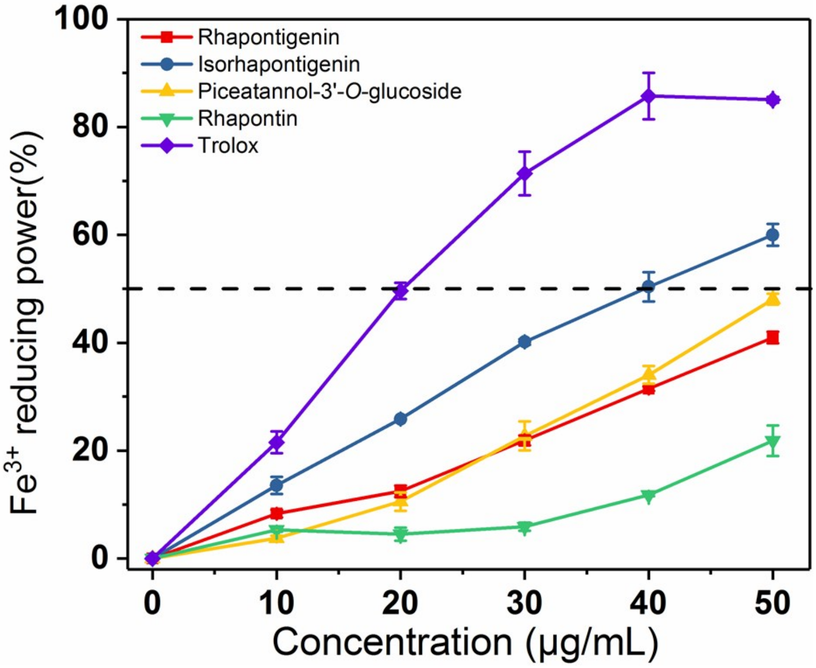

Fig. S3 Dose response curves in the Fe^{3+}-reducing power spectrophotometric analysis.

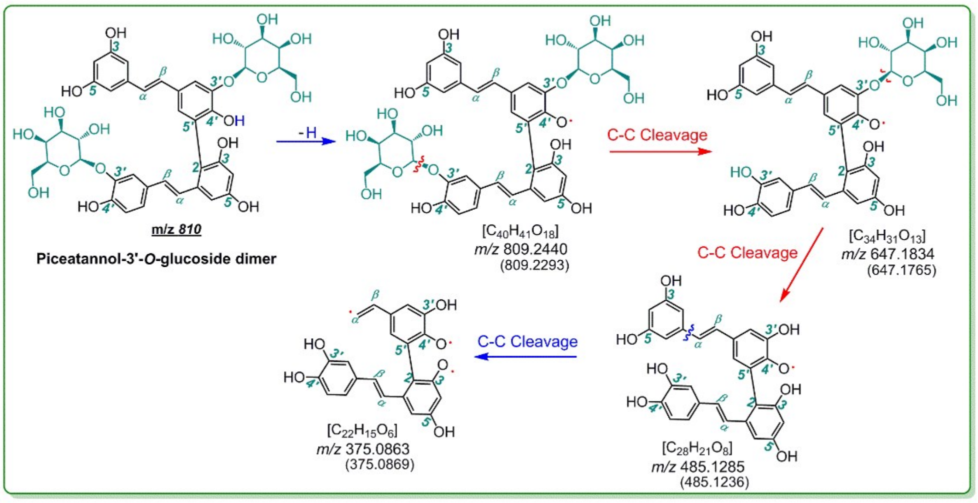

Fig. S4 MS elucidation of piceatannol-3'-Oglucoside dimer

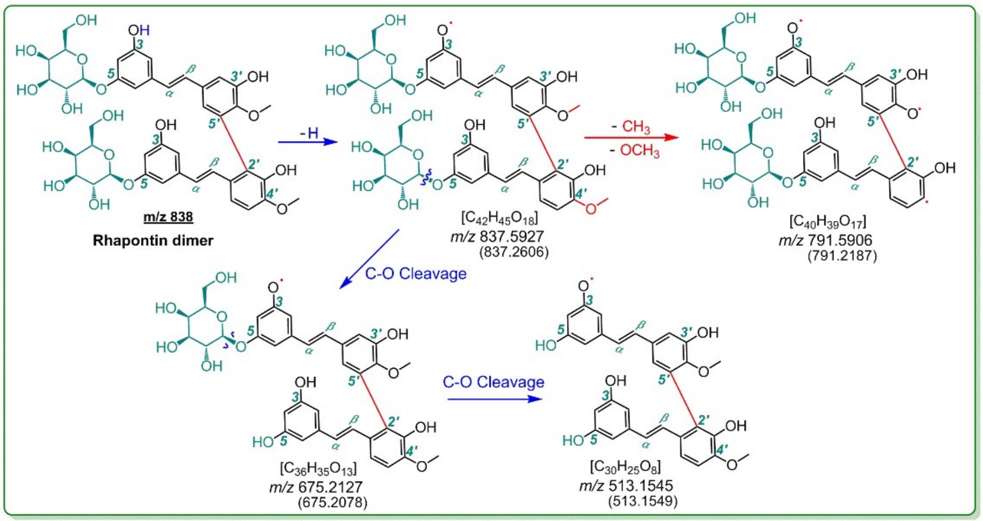

Fig. S5 MS elucidation of rhapontin dimer

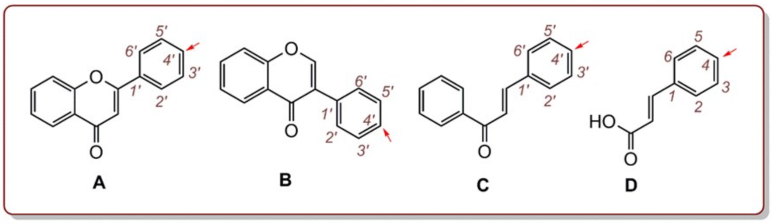

Fig. S6 Skeletons of part phenolic families: A, flavonoid, B, isoflavonoid, C, Chalcone, D, Cinnamic acid derivatives (The arrow indicated the so-called “preferential position”)
